# Telehealth Use Among Older Adults Receiving Home- and Community-Based Services

**DOI:** 10.1093/geroni/igaf122.1194

**Published:** 2025-12-31

**Authors:** Dana Urbanski, Romil Parikh, Jack Wolf, Benjamin Langworthy, Chanee Fabius, Janette Dill, Eric Jutkowitz, Tetyana Shippee

**Affiliations:** Indiana University Bloomington, Bloomington, Indiana, United States; University of Minnesota School of Public Health, Minneapolis, Minnesota, United States; University of Minnesota School of Public Health, Minneapolis, Minnesota, United States; University of Minnesota School of Public Health, Minneapolis, Minnesota, United States; Johns Hopkins University, Baltimore, Maryland, United States; University of Minnesota School of Public Health, Minneapolis, Minnesota, United States; University of Minnesota, Minneapolis, Minnesota, United States; University of Minnesota, Minneapolis, Minnesota, United States

## Abstract

Telehealth was essential for maintaining care continuity during the COVID-19 pandemic, leading to its rapid expansion and adoption in the United States. Since then, telehealth has been increasingly viewed as a strategy to reduce health disparities among vulnerable populations. However, data on telehealth use among socially and financially vulnerable older adults are limited, leaving unanswered questions about the role of telehealth in promoting health equity for older Americans. This study examined individual and structural factors associated with post-pandemic telehealth use among older recipients of publicly funded home- and community-based services (HCBS; an indicator of social and financial vulnerability). Using cross-sectional data from the 2021–2022 National Core Indicators-Aging and Disability Adult Consumer Survey, the analytic sample included 3,680 HCBS users aged ≥ 65 years without intellectual or developmental disability, 40% of whom used telehealth. Complete-case multivariable logistic regression, adjusting for sociodemographic and health-related factors with state-level random intercepts, revealed significantly lower odds of telehealth use associated with older age, male gender, Black race, non-metropolitan residence, and receipt of Older Americans Act services. Individuals with dementia had higher odds of telehealth use (OR, 1.33, p < .05). Home internet access was strongly associated with telehealth use (OR, 2.51, p < .001). Follow-up bivariate analyses showed younger age, female gender, and White race were associated with higher internet access. These findings highlight differences in telehealth use among older HCBS users associated with both individual and structural factors, offering valuable insights for efforts aimed at addressing barriers to telehealth adoption among vulnerable older adults.

